# The impact of systemic administration of polyphenols on periodontitis associated with diabetes mellitus: a systematic review

**DOI:** 10.2340/aos.v83.40484

**Published:** 2024-05-03

**Authors:** Kornelija Rogalnikovaite, Auste Antipoviene, Asta Burbulyte, Egle Aida Bendoraitiene

**Affiliations:** a Department of Preventive and Paediatric Dentistry, Lithuanian University of Health Sciences, Kaunas, Lithuania; b Department of Dental and Oral Pathology, Lithuanian University of Health Sciences, Kaunas, Lithuania; c Library and Information Centre, Information Services Division, Lithuanian University of Health Sciences, Kaunas, Lithuania

**Keywords:** Diabetes mellitus, periodontitis, polyphenols, inflammation

## Abstract

**Objective:**

The aim of this work was to explore the potential of polyphenol supplement consumption in enhancing the treatment of periodontitis and diabetes mellitus in both diabetic animals and humans.

**Materials and methods:**

A comprehensive search across eight databases (MEDLINE, EBSCO, Taylor & Francis, PRIMO, Web of Science, Wiley Online Library, ScienceDirect, and SAGE Journals) and two registers (ClinicalTrials.gov and Cochrane Library Trials) was conducted. Methodological quality assessment employed the Cochrane Collaboration Risk of Bias Assessment Tool for randomised controlled trials and the Systematic Review Centre for Laboratory Animal Experimentation Risk of Bias Tool for experimental animal studies.

**Results:**

Ten articles meeting inclusion criteria were identified. Three clinical studies demonstrated significant reductions in probing depth (PD) and clinical attachment loss (CAL). Ginger supplementation showed a decrease in CAL (–0.57 ± 0.50 vs. –0.14 ± 0.35, *p* = 0.003) and PD (–0.52 ± 0.51 vs. –0.19 ± 0.51, *p* = 0.04), while resveratrol supplementation exhibited a reduction in PD (–1.1 ± 0.58 vs. –0.6 ± 0.47, *p* < 0.001). Additionally, cranberry juice supplementation led to a decrease in PD (–0.56 ± 0.03, *p* < 0.001). However, there was no significant improvement in inflammation status. Although polyphenol supplementation did not impact fasting blood glucose levels, it did result in improved insulin resistance (3.66 ± 0.97 vs. 4.49 ± 1.56, *p* = 0.045). In diabetic animals, six studies reported a significant reduction (*p* < 0.05) in bone loss along with marked improvements in inflammation status.

**Conclusions:**

Despite the promising results observed in the included studies, the overall evidence supporting the positive effects of polyphenols on periodontal and diabetes mellitus status, along with their anti-inflammatory properties, remains inadequate.

## Introduction

Periodontitis is a chronic inflammatory disease that leads to the destruction of the periodontal ligament, connective tissue, and alveolar bone. It affects up to 62% of the adult population [1]. In addition to tissue destruction and tooth loss, periodontal disease and the presence of associated pathogens such as *Porphyromonas gingivalis*, *Aggregatibacter actinomycetemcomitans*, and *Fusobacterium nucleatum* have now been linked to a wide spectrum of extra-oral diseases. These include Alzheimer’s disease [2], diabetes mellitus (DM) [3], cardiovascular disease [4], colorectal cancers [5], inflammatory bowel disease [6], rheumatoid arthritis [7], non-alcoholic fatty liver disease [8], and obesity [9]. The periodontal microbiota exerts an impact on systemic diseases through two primary and potentially synergistic mechanisms: direct disease-promoting effects resulting from the migration of oral bacteria to distant locations and a variety of indirect effects due to the presence of imbalanced oral microbial communities in the mouth [10]. The relationship between periodontal disease and other inflammatory conditions is not fully understood, and ongoing debates exist, considering its bidirectional nature [11].

While changes in the oral microbiota play a specific role in the development of periodontitis, its aetiology is multifactorial [12]. The host response to microbial invasion involves the upregulation of proinflammatory cytokines, matrix metalloproteinases, and reactive oxygen species (ROS) [13, 14]. ROS help to eliminate periodontal pathogens, but if overstimulated, they can lead to connective tissue destruction and periodontal attachment loss due to oxidative stress – an imbalance between the production and elimination of reactive oxygen/nitrogen species (ROS/RNS) [14]. Oxidative stress is a common characteristic of chronic periodontitis and other systemic inflammatory diseases, such as obesity or DM [13, 15, 16].

Considering oxidative stress as a therapeutic target for chronic periodontitis, antioxidants are believed to be beneficial as adjuncts to periodontal treatment. Polyphenols, a class of compounds found in many plant foods, play a potential pro-oxidant role and protect our bodies from severe cellular oxidative stress [17]. Their antioxidant effect makes them beneficial in preventing and treating several inflammatory diseases, such as type 2 diabetes mellitus (T2DM) [18] and Alzheimer’s disease [19]. Given the well-analysed relationship between periodontal diseases and DM, particularly the high prevalence in diabetic patients [20, 21], the aim of this systematic review is to investigate whether polyphenol supplement consumption could improve the treatment of periodontitis and DM both diabetic animals and humans.

## Materials and methods

### Protocol and registration

The systemic analysis review report adhered to the Preferred Reporting Item for Systematic Review and Meta-Analyses (PRISMA) statement [22]*.* The review was registered on PROSPERO system under number CRD42023402629.

### Focus question

The following focus question was formulated using the Population, Intervention, Comparison, and Outcome (PICO) framework (Table 1): Is there evidence to suggest that the administration of polyphenols as a systemic treatment can influence the regression of periodontitis and improve the diabetic condition in individuals and animal models with DM?.

**Table 1:** PICO search strategy.

Framework item	Description
Population	Diabetic patients and animals (mice and/or rats) with periodontitis
Intervention	Systemic administration of various polyphenols and its derivates for at least 28 days
Comparison	Systemic administration of placebo solution for at least 28 days
Outcome	Polyphenol supplementation demonstrates several positive effects, including improvement in clinical periodontal parameters, reduction in the expression and levels of inflammatory mediators, and effective control of hyperglycaemia in both patients and mice and/or rats with DM, as compared to the control group

PICO: Population, Intervention, Comparison, and Outcome.

### Information sources and search strategy

An information professional (A.B.) conducted an electronic search in a total of eight databases and two registers from October 27, 2022 to December 1, 2022. The search was performed in the following sources: MEDLINE (PubMed), EBSCO, Taylor & Francis, PRIMO, Web of Science, Wiley Online Library, ScienceDirect, SAGE Journals, ClinicalTrials.gov, and Cochrane Library Trials. To minimise the inclusion of irrelevant articles, an additional filter of ‘dentistry’ was applied specifically to the Wiley Online Library database. The search utilised Medical Subject Headings (MeSH) terms, namely ‘polyphenols’, ‘periodontitis’, and ‘periodontal diseases’ combined using the Boolean operator ‘AND’. The search strategy is presented in Table 2.

**Table 2:** Electronic search strategy.

Search date	Database/register	Keywords	Records
2022–12-01	MEDLINE (PubMed)	(‘polyphenols’ [All Fields] AND ‘periodontitis’ [All Fields])	197
		(‘polyphenols’ [All Fields] AND ‘periodontal diseases’ [All Fields])	194
2022–12-01	EBSCO	(Polyphenols AND periodontitis)	93
2022–12-01	Taylor & Francis	(Polyphenols AND periodontitis)	86
2022–12-01	PRIMO	(Polyphenols AND periodontitis)	153
2022–12-01	Web of Science	(Polyphenols AND periodontitis)	126
2022–12-01	Wiley Online Library	(Polyphenols AND periodontitis)	313
2022–12-01	ScienceDirect	(Polyphenols AND periodontitis)	548
2022–12-01	SAGE Journals	(Polyphenols AND periodontitis)	18
2022–12-01	ClinicalTrials.gov	(Polyphenols AND periodontitis)	3
2022–12-01	Cochrane Library Trials	(Polyphenols AND periodontitis)	11

### Selection of studies

The titles and abstracts of all the studies identified, as well as the full text of potentially eligible investigations, were independently screened by two review authors (K.R. and A.A.) using predefined inclusion and exclusion criteria. In cases where there was a disagreement regarding the eligibility of articles, a third reviewer (E.A.B.) was involved to resolve any conflicts through collaboration.

### Inclusion and exclusion criteria

The following inclusion criteria were applied during the screening process:

- Randomised controlled trials (RCTs) and experimental animal studies
- T2DM patients with periodontitis
- Mice and/or rats with type 1 diabetes mellitus (T1DM) or T2DM induced using streptozotocin solution, with experimental periodontitis induced by the ligation method
- Systemic administration of polyphenols for a minimum duration of 28 days
- Assessment of changes in clinical periodontal parameters, including periodontal bone level (PBL), alveolar bone loss (ABL), probing depth (PD), and/or clinical attachment loss (CAL)
- Analysis of changes in the expression and levels of inflammatory mediators, fasting blood glucose (FBG), glycosylated haemoglobin levels (HbA1c), and glycaemic index
- Studies published from January 1, 2012
- Studies written in the English language
- Full-text access to the articles

The following exclusion criteria were also applied:

- Clinical or preclinical trials without a control group
- Studies involving systemically healthy patients with periodontitis
- Experimental periodontitis studies in mice and/or rats without a DM model
- Local administration of polyphenols
- The duration of systemically administered polyphenols was less than 28 days
- Systematic reviews, letters, dissertations, case reports, conference abstracts, and theses

### Data extraction

A narrative synthesis was conducted by two independent review authors, K.R. and A.A., using studies that met the inclusion criteria. The studies were categorised into two groups: investigations reporting the effect of systemic administration of polyphenols on periodontal tissues, inflammation markers and diabetes condition in patients with DM and separately in diabetic animals. The pertinent information was synthesised by tabulating the data according to (1) authors and year, (2) study design, (3) study population, (4) interventions, (5) experimental period, (6) analysed parameters, and (7) results.

### Risk of bias assessment

During the data extraction process, the methodological quality of the included studies was assessed by two independent reviewers (K.R. and A.A.). Similarly, in situations where disagreements arose regarding the risk of bias, a third reviewer (E.A.B.) was engaged to facilitate consensus and resolve any conflicts through collaborative discussion.

For RCTs, the risk of bias was analysed using the Cochrane Collaboration Risk of Bias Assessment Tool [23]. The following parameters were evaluated to determine the risk of bias: (1) random sequence generation, (2) allocation concealment, (3) blinding of participants and personnel, (4) blinding of outcome assessment, (5) incomplete outcome data, (6) selective reporting, and (7) other biases. The risk of bias was classified as low (+), high (–), or unclear (×) for each parameter. Studies were categorised as having a low risk of bias if all criteria were met, unclear if one criterion was not met or the risk of bias was unclear for two criteria and high if two or more criteria were not met.

For the included animal studies, the risk of bias was assessed using the Systematic Review Centre for Laboratory Animal Experimentation Risk of Bias (SYRCLE’s RoB) Tool [24]. This tool includes 10 entries related to six types of bias: (1) selection bias, (2) performance bias, (3) detection bias, (4) attrition bias, (5) reporting bias, and (6) other biases. The risk of bias was classified as ‘high’ (–), ‘low’ (+), or ‘unclear’ (?) if there were insufficient details reported to properly assess the risk of bias.

## Results

### Study selection

**Figure 1:**
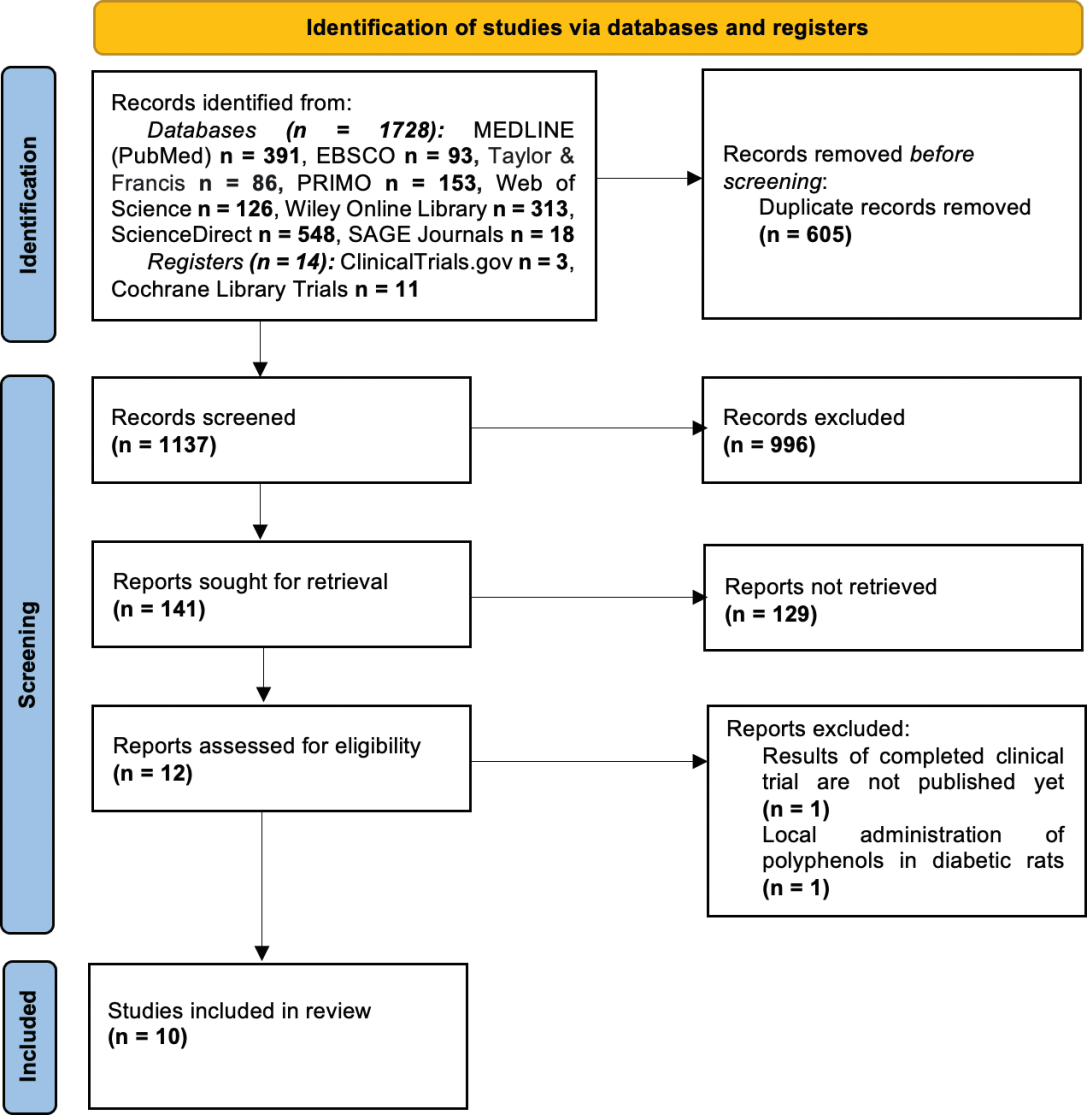
PRISMA flow diagram.

The search strategy in electronic databases and registers initially identified 1,742 investigations. All records were exported to RefWorks® (ProQuest CSA, Bethesda, MD, United States) bibliography and citation manager. After removing duplicates, a total of 1,137 articles remained for screening. Based on the titles, 996 articles were excluded. Following this exclusion, a more detailed screening was conducted on the remaining 141 articles’ abstracts. From this screening, 12 publications were identified as meeting the criteria for full-text analysis. Two of them were further excluded for the following reasons: one study administered polyphenols as a local application in diabetic rats [25], and another study’s results were not yet available as it was a completed clinical trial registered on the Cochrane Central Register of Controlled Trials [26]. Finally, a total of 10 articles were included in the systematic review. The study selection process is presented in Figure 1.

### Risk of bias assessment

**Figure 2:**
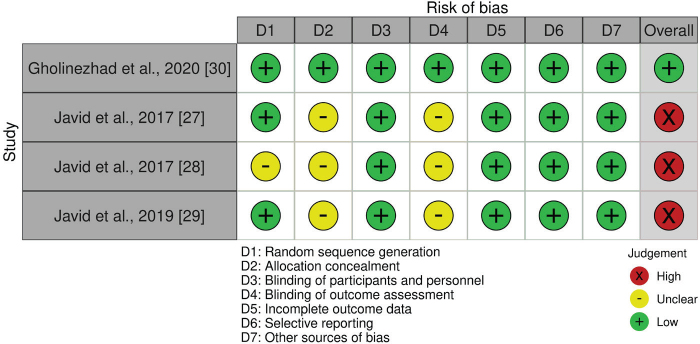
Assessment of risk of bias for clinical studies.

After assessing the methodological quality of RCTs, it was identified that three clinical trials [27–29] were at a high risk of bias. The study conducted by Gholinezhad et al. [30] was rated as having high quality based on the evaluation criteria. A detailed assessment of the risk of bias is presented in Figure 2.

**Figure 3:**
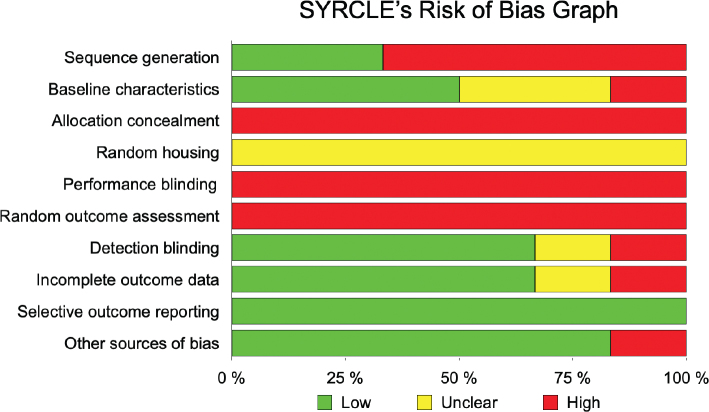
A summary of risk of bias for each item in the SYRCLE.

Regarding the preclinical studies with animals, the quality assessment revealed that all of them were classified as high risk in the domains of ‘allocation concealment’, ‘random outcome assessment’ and ‘performance blinding’. Additionally, there was insufficient information provided regarding animal housing during the experiments. For the entry ‘selective outcome reporting’, all expected outcomes were reported, indicating a low risk of bias. Most of the studies showed a low risk of bias for the items ‘detection blinding’, ‘incomplete outcome reporting’, and ‘other biases’, with 67% and 83% of studies demonstrating low risk, respectively. These findings are summarised in Table 3 and Figure 3.

**Table 3:** Assessment of methodological quality of preclinical studies.

Author, year	Sequence generation	Baseline characteristics	Allocation concealment	Random housing	Performance blinding	Random outcome assessment	Detection blinding	Incomplete outcome data	Selective outcome reporting	Other sources of bias
Alpan et al., 2022 [31]	+	?	–	?	–	–	+	+	+	+
Catanzaro et al., 2018 [32]	–	+	–	?	–	–	+	+	+	+
Cirano et al., 2021 [33]	–	+	–	?	–	–	+	+	+	+
Gennaro et al., 2015 [34]	–	–	–	?	–	–	?	–	+	+
Toker et al., 2018 [35]	–	?	–	?	–	–	+	+	+	–
Zhen et al., 2015 [36]	+	+	–	?	–	–	–	?	+	+

+: low risk; ?: unclear risk; –: high risk.

### Characteristics of included studies

The characteristics of the included studies are described in Tables 4 and 5. This systematic review included a total of four RCTs [27–30] and six preclinical animal studies [31–36]. The study population consisted of 169 patients and 216 animals, specifically 196 Wistar rats and 20 C57BLKS/J-db/db mice. The experimental period in clinical trials ranged from 4 [27, 29] to 8 [28, 30] weeks, while in preclinical studies, it ranged from 28 [36] to 90 [32, 34] days. The administered polyphenols varied among the studies, with the most used ones being resveratrol [27, 29, 33, 36], ginger [30], cranberry juice [28], green tea extract [32, 34], taxifolin [31], and grape seed proanthocyanidin extract [35]. In the case of diabetic patients with periodontitis, polyphenol supplementation was administered orally as tablets, except for cranberry juice, which was consumed in liquid form. Rats and mice received different polyphenols via gavage [31, 33, 35, 36] or *ad libitum* [32, 34]. DM was induced in animals by intraperitoneal injection of streptozotocin, with concentrations ranging from 47 [32] to 60 mg/kg [33, 35]. One study by Zhen et al. [36] utilised C57BLKS/J-db/db mice, which are a model of T2DM. Experimental periodontitis was induced by ligation with 4-0 silk sutures [31, 35] or cotton ligatures [33, 36]. In the study by Zhen et al. [36], cotton ligatures were pre-soaked in a medium with *P. gingivalis*. However, in the studies by Catanzaro et al. [32] and Gennaro et al. [34], no ligation was performed. Given the fact that DM can worsen periodontal diseases, the authors of the included studies analysed the possible changes in periodontal tissues caused by hyperglycaemia. PD was evaluated in three clinical trials [27, 28, 30], while CAL was assessed in two of four selected studies [29, 30]. In addition, the majority of included articles focused on total antioxidant capacity [29, 30], HbA1c [28, 30] and FBG [27, 28, 30]. Furthermore, Javid et al. [27] measured insulin levels and utilised the homeostasis model to assess insulin resistance. In all preclinical studies [31–36], ABL or PBL was measured. Some investigations also evaluated changes in the expression of cytokines, inflammatory markers, or oxidative stress markers, with receptor activator of nuclear factor kappa-B ligand (RANKL) [31, 34], osteoprotegerin (OPG) [33, 34], interleukins (ILs) [33, 34, 36], and tumour necrosis factor alpha (TNF-α) [34, 36] being among the markers of interest. Concerning diabetes condition, blood glucose levels [31, 33, 35, 36] and the glycaemic index [32, 34] were assessed in diabetic animals.

**Table 4:** Characteristics of included clinical trials.

Author	Gholinezhad et al. [30]	Javid et al. [27]	Javid et al. [28]	Javid et al. [29]
Publication year	2020	2017	2017	2019
N total N intervention N placebo	42 21 21	43 21 22	41 I1 = 10; I2 = 9; I3 = 10 12	43 21 22
Study design	RCT	RCT	RCT	RCT
Intervention group	Ginger supplement 2 g/d as four tablets ( 2× daily 2 tablets) + NST	480 mg/d resveratrol (2 pills) + NST	I1 omega-3, 1g/2 times a day I2 cranberry juice 200 ml 2 times a day I3 cranberry juice enriched with omega-3,200 ml + 1 g omega-3 2 times a day	480 mg/d resveratrol (2 pills) + NST
Control group	Placebo + NST	Placebo capsules (2 pills) + NST	NST	Placebo capsules (2 pills) + NST
Type of intervention	Ginger supplement + NST	Resveratrol + NST	Omega-3 or cranberry juice or omega-3 with omega-3 + NST	Resveratrol + NST
Duration of intervention	8 weeks	4 weeks	8 weeks	4 weeks
Periodontal status results	↓ΔCAL (−0.57 ± 0.50 vs. −0.14 ± 0.35, *p* = 0.003); ↓ΔPD (−0.52 ± 0.51 vs. −0.19 ± 0.51, *p* = 0.04)	↓ΔPD (−1.1 ± 0.58 vs. −0.6 ± 0.47, *p* < 0.001)	↓ΔPD (−0.77 ± 0.3 vs. −0.56 ± 0.03 vs −1.08 ± 0.49 vs. −0.9 ± 4.9, *p* < 0.001)	ΔCAL not evaluated;post-intervention CAL (2 ± 0.4 vs. 2.2 ± 0.5, *p* = 0.06)
Inflammation status results	ΔTAC (−0.006 ± 0.09 vs. 0.014 ± 0.03, *p* = 0.33); ΔMDA (−0.13 ± 8.66 vs. −3.37 ± 7.16, *p* = 0.19)	−	−	ΔIL-6, ΔTNF-α, ΔTAC not evaluated; post-intervention IL-6 (1.58 ± 1.06 vs. 1.85 ± 0.59, *p* = 0.3); TNF-α (10.33 ± 0.66 vs. 10.57 ± 0.68, *p* = 0.25); TAC (1.35 ± 0.62 vs. 1.02 ± 0.43, *p* = 0.05)
Diabetic status results	↓ΔHbA1C (−0.75 ± 1.17 vs. −0.16 ± 0.44, *p* = 0.04); ΔFBG (−2.57 ± 27.05 vs. −19.04 ± 39.49, *p* = 0.12)	FBG (9.19 ± 2.78 vs. 7.9 ± 2.12, *p* = 0.097); ↓insulin (10.42 ± 0.28 vs. 10.92 ± 0.9, *p* = 0.02), ↓HOMA-IR (3.66 ± 0.97 vs 4.49 ± 1.56, *p* = 0.045)	ΔFBG (−31.40 ± 64.73 vs.−28.00 ± 46.89 vs. −3.11 ± 69.29 vs. 5.57 ± 38.87, *p* = 0.492);ΔHbA1C (−0.29 ± 0.67 vs. −0.87 ± 0.86 vs. −0.25 ± 0.51 vs. −0.40 ± 0.42, *p* = 0.2)	−
Conclusions	Positive effect on periodontal and diabetes status in patients with DM.	Positive effect on periodontal and diabetes status in patients with DM.	Positive effect on periodontal status in patients with DM.	No positive effect on periodontal status in patients with DM.

RCT: randomised controlled trial; I: intervention; NST: non-surgical periodontal treatment; ΔCAL: changes in clinical attachment loss; ΔPD: changes in pocket depth; ΔHbA1c: changes in glycosylated haemoglobin levels; DM: diabetes mellitus; HOMA-IR: insulin resistance; ΔFBG: changes in fasting blood glucose; ΔTAC: changes in total antioxidant capacity; ΔMDA: changes in malondialdehyde; TNF-α: tumour necrosis factor; IL-6: interleukin 6; ↑/↓: statistically significant decrease, resp. increase in comparison of test/control group.

**Table 5:** Characteristics of included animal studies.

Author, year	Alpan et al., 2022 [31]	Catanzaro et al., 2018 [32]	Cirano et al., 2021 [33]	Gennaro et al., 2015 [34]	Toker et al., 2018 [35]	Zhen et al., 2015 [36]
Study design	Experimental animal study	Experimental animal study	Experimental animal study	Experimental animal study	Experimental animal study	Experimental animal study
N total N intervention N control	30 I1 = 10; I2 = 10 C = 10	50 I = 25 C = 25	54 I1 = 13; I2 = 14 C1 = 14; C2 = 15	40 I = 20 C = 20	22 I1 = 8; I2 = 8 C = 6	20 I = 10 C = 10
Intervention group	I1 Taxi-5 I2 Taxi-10 29 days	GT *ad libitum* 90 days	10 mg/kg of RSV I1 – DM+RSV I2 – DM+INS+RSV 30 days	GT *ad libitum* 90 days	I1 GSPE-100 I2 GSPE-200 30 days	20 mg/kg of RSV 4 weeks
Control group	Untreated mice with DM and PE	Distilled water *ad libitum*	C1 – DM+PL C2 – DM+INS	Distilled water *ad libitum*	Untreated mice with DM and PE	Untreated mice with DM and PE
Periodontal status results	↓bone loss (*p* < 0.05);↑ BMP-2 (1.14 ± 0.01 vs. 2.00 ± 0.81 vs. 2.85 ± 0.37, *p* < 0.05); ↑ type I collagen (0.42 ± 0.20 vs. 1.85 ± 0.89 vs. 2.14 ± 0.89, *p* < 0.05); ↑ ALP (0.42 ± 0.20 vs. 1.85 ± 0.69 vs. 2.71 ± 0.48, *p* < 0.05); ↑ OCN (1.42 ± 0.20 vs. 2.00 ± 0.57 vs. 2.28 ± 0.95, *p* < 0.05); ↑ BCL-2 (0.14 ± 0.01 vs. 1.85 ± 0.89 vs. 2.28 ± 0.95, *p* < 0.05)	↓bone loss PBL (1.34 ± 0.21 vs. 0.93 ± 0.11, *p* < 0.05)	I1 ↓bone loss (*p* < 0.05)	↓bone loss (ABC-CEJ 468.4 ± 61.4 vs. 341.5 ± 26.1, *p* < 0.05)	↓bone loss in I2 (*p* < 0.05), ↑osteoblast activity (*p* < 0.05)	↓bone loss (*p* < 0.05)
Inflammation status results	↓Bax (2.28 ± 0.48 vs. 1.42 ± 0.53 vs. 1.14 ± 0.69, *p* < 0.05); ↓RANKL in I2 (1.85 ± 0.69 vs 0.28 ± 0.18, *p* < 0.05)	–	I2 ↓ IL-1β (*p* < 0.05), ↓ IL-6 (*p* < 0.05), ↑SOD (*p* < 0.05), I1 ↓ NADPH oxidase (*p* < 0.05), I1 ↓OPG (6.143 ± 4.552 vs. 1.098 ± 1.646, *p* < 0.05), I1 ↑SIRT mRNA(28.127 ± 27.094 vs. 4.590 ± 5.712, *p* < 0.05), I1↓RANKL(42.653 ± 24.813 vs. 49.616 ± 28.662, *p* < 0.05)	↓ IL-10 (1740.9 ± 38 vs. 955.1 ± 114.4, *p* < 0.05), ↑OPG(2211.5 ± 74.6 vs 1791.6 ± 78.3, *p* < 0.05), ↓ RANKL (1046.7 ± 31.6 vs 1653.6 ± 41.9, *p* < 0.05, ↓TNF-α (1,141 ± 71 vs. 1,653 ± 104, *p* < 0.05), ↑RUNX-2 (*p* < 0.05)	↓ inflammatory cells (*p* < 0.05), ↓(MMP)-8 (200.07 ± 29.00 vs. 91.77 ± 48.00 vs 52.72 ± 24.39, *p* < 0.05), ↓(HIF)-1α (150.50 ± 44.03 vs. 81.98 ± 44.19 vs. 74.13 ± 28.23, *p* < 0.05)	↓ IL-1β, IL-6, IL-8 (*p* < 0.05), ↓TNF-α (*p* < 0.05), ↓TLR4 (*p* < 0.05)
Diabetes status results	Blood glucose (444.71 ± 32.73 vs. 472.85 ± 14.97 vs. 433.71 ± 31.62, *p* > 0.05)	↓Glycaemic level (284.4 vs. 357.2, *p* < 0.05)	I1 ↓ glucose level (282.07 ± 7 vs. 445.92 ± 7, *p* < 0.05)	↓Mean glucose level (281.2 ± 21.4 vs. 361.7 ± 16.0, *p* < 0.05)	Blood glucose (444.0 ± 36.0 vs. 447.0 ± 36.0 vs 451.0 ± 35.0, *p* > 0.05)	↓Fast plasma glucose (*p* < 0.05)

I: intervention; C: control; Taxi-5: taxifolin 5 mg/kg; Taxi-10: taxifolin 10 mg/kg; DM: diabetes mellitus; PE: experimental periodontitis; BMP-2: bone morphogenic protein; OCN: osteocalcin; ALP: alkaline phosphatase; BAX: pro-aptotic Bax protein; Bcl-2B: protein regulator of apoptosis; RANKL: receptor activator of nuclear factor kappa-B ligand; GT: green tea; PBL: periodontal bone level; RSV: resveratrol; PL: placebo; INS: insulin therapy; IL-1β: interleukin 1β; IL-6: interleukin 6; IL-7: interleukin 7; SOD: superoxide dismutase 1; NADPH oxidase: nicotinamide adenine dinucleotide phosphatase oxidase; OPG: osteoprotegerin; SIRT 1: Sirtuin 1; ABC: alveolar bone crest; CEJ: cementoenamel junction; RUNX-2: runt-related transcription factor 2; IL-10: interleukin 10; TNF-α: tumour necrosis factor alfa; GSPE-100: grape seed proanthocyanidin extract 100 mg/kg/day; GSPE-200: grape seed proanthocyanidin extract 200 mg/kg/day; (MMP)-8: matrix metalloproteinase; (HIF)-1α: hypoxia inducible factor; IL-8: interleukin 8; TLR4: Toll-like receptor 4; IFN-γ: interferon-gamma; ↑/↓: statistically significant decrease, resp. increase in comparison of test/control group.

## Discussion

The current systematic review is primarily focused on assessing the supplementary systemic administration of polyphenols in individuals and mice with DM. It is widely acknowledged that polyphenols can exert localised effects in the treatment of periodontitis by promoting beneficial oral bacterial communities and suppressing inflammation [37–42]. Furthermore, these compounds can also have downstream effects or function through phenolic metabolites to mitigate inflammatory and metabolic issues linked to diabetes [37]. Local applications of polyphenols have shown favourable outcomes in periodontal treatment, demonstrating antibacterial and immunomodulatory properties [38, 39]. Das et al. [40] illustrated that grape seed extract, when used in conjunction with scaling and root planing, significantly reduced measures of periodontal disease, including PD and relative attachment level (RAL). Similarly, Chava et al. [41] reported that a thermo-reversible green tea catechin gel reduced PD and RAL, Elavarasu et al. [42] demonstrated that curcumin strips acted as antioxidants, lowering superoxide dismutase levels. However, it is important to notice that localised applications of polyphenols do not alter the host immune response to chronic bacterial infection as does systemic consumption [43].

Polyphenols have been studied for their beneficial effects on various diseases due to their anti-inflammatory and antioxidant properties. Chedea et al. [44] raised the question of whether grape pomace polyphenols could be used as an alternative or adjuvant treatment to non-steroidal anti-inflammatory drugs to minimise side effects. Grape pomace was found to have promising potential in reducing oxidative stress and inflammation markers, as evidenced in various *in vivo* and *in vitro* studies. The synthesis of various polyphenols is believed to have a synergistic effect and may be more effective than single compound polyphenols, warranting further investigation. It is worth noting that other natural micronutrients, such as carotenoids and vitamins, also possess antioxidant properties and function as anti-inflammatory agents. Their actions may complement and synergise with those of polyphenols, both in dietary contexts and as potential supplementary therapeutic agents [37]. Considering those drawbacks of systemic polyphenols administration, it may have limited effectiveness due to the unfavourable pharmacokinetic and pharmacodynamic properties. Despite their significant potential, polyphenolic compounds are associated with limited bioavailability, primarily owing to low solubility, poor stability in the gastrointestinal tract, low intestinal permeability, and an extremely short plasma half-life [43].

Both DM and periodontal disease are associated with inflammatory processes and oxidative stress. The antioxidant defence system can be partially interrupted due to excessive production of free radicals, leading to altered inflammatory responses in individuals with underlying medical conditions, particularly those with diabetes. This suggests the potential benefits of additional anti-inflammatory treatment [20]. Various host immune system modulators could be used in addition to non-surgical periodontal treatment. A systematic review by Corbella et al. [45] discovered very low evidence regarding the adjunctive use of sub-antimicrobial doses of doxycycline, melatonin, and the combination of omega-3 and low-dose aspirin (in T2DM patients) to non-surgical periodontal treatment in modulating host response and improving PD and/or CAL. However, the evidence remains limited, and official recommendations for using host modulators in addition to periodontal treatment are lacking.

In our systematic review, three [27, 28, 30] of four human studies demonstrated a significant positive effect on periodontal status in patients with DM who consumed polyphenol supplements, although only one study [30] was evaluated as of high quality. In comparison to the control group, Gholinezhad et al. [30] observed a significant reduction in CAL and PD in 4 and 2.7 times, as well as Javid et al. [27] noticed an improvement in PD in 1.8 times. It is interesting that Javid et al. [28] found that omega-3 fatty acid, which acts as a host modulator exhibited a higher reduction in PD compared to cranberry juice. However, Javid et al. [29] reported no statistically significant reduction in CAL between the two groups post-intervention, even though CAL decreased significantly in both the intervention and control groups.

In spite of the fact that inflammation status in diabetic patients needs further investigations, the researchers observed positive effect while consuming polyphenol supplements. Ginger has been studied for its potential anti-inflammatory properties, and some authors have suggested that ginger extracts may inhibit the formation of inflammatory compounds, including prostaglandins and leukotrienes [46]. Gholinezhad et al. [30] highlighted significantly decreased levels of malondialdehyde and improved TAC in the intervention group, although these changes in inflammation markers did not reach statistical significance between the two groups post-intervention. Indeed, the findings suggest that resveratrol could have a beneficial impact on inflammatory processes. Its ability to activate antioxidant enzymes and suppress the release of proinflammatory cytokines such as TNF-α, IL-1β, IL-6, IL-10 and interferon-beta (IFN-β) in a wide range of tissues is of particular interest [47]. The findings from Javid et al. [29] suggest that resveratrol supplementation, when combined with non-surgical periodontal treatment, led to a significant decrease in IL-6 levels. However, when comparing the intervention group to the control group, no significant differences were observed in main inflammation markers such as IL-6, TNF-α, and TAC. The researchers highlighted an important consideration – while inflammatory markers reduced in response to common treatment, factors such as genetics, epigenetics, susceptibility, response variations, and other environmental circumstances could potentially interfere with the results. Consequently, they emphasised that the impact of polyphenols on inflammation degree cannot be conclusively determined.

The authors also reported improved DM parameters: Gholinezhad et al. [30] showed significantly decreased HbA1c and FBG levels in the intervention group; however, intergroup comparison revealed statistically significant improvement only in HbA1c values. Javid et al. [28] demonstrated significantly decreased HbA1c while consuming omega-3 fatty acid and cranberry juice, but overall changes in FBG and HbA1c levels were not significantly different between groups at baseline and post-intervention. Javid et al. [27] reported significantly improved insulin resistance, suggesting that polyphenols may act on common inflammatory processes in organisms. It has been established that dietary polyphenols play a significant role in treating type T2DM through insulin-dependent mechanisms. Curcumin has been shown to lower blood sugar and HbA1c. In addition, this polyphenol could enhance insulin sensitivity [48]. Likewise, resveratrol has been demonstrated to enhance glucose uptake and metabolism, promote pancreatic beta-cell protection, and improve insulin resistance [49].

Given the limited evidence from human studies, animal studies were also included in the review, revealing consistent positive outcomes. Across all investigations, the consumption of polyphenol substitutes resulted in a significant reduction in bone loss compared to control groups. Cirano et al. [33] demonstrated that resveratrol, either alone or combined with insulin, markedly decreased alveolar bone loss in diabetic animals compared to those receiving a placebo solution. Zhen et al. [36] reported a similar trend, showing that resveratrol treatment reduced alveolar bone loss in db/db mice with experimental periodontitis. Gennaro et al. [34] observed significantly diminished bone loss in diabetic rats ingesting green tea compared to those consuming water. Additionally, Catanzaro et al. [32] corroborated this observation, reporting that diabetic mice consuming green tea experienced less bone loss compared to those consuming water. Noteworthy findings emerged from the investigations by Alpan et al. [31] and Toker et al. [35], where different concentrations of taxifolin and grape seed proanthocyanidin were employed. Polyphenol administration significantly decreased alveolar bone loss, with no significant differences observed at higher concentrations. Nevertheless, Toker et al. [25] highlighted that polyphenol supplementation increased osteoblast activity. In the context of diabetes, which exacerbates pathological processes, including increased bone resorption and inhibited coupled bone formation, leading to accelerated bone loss, polyphenolic compounds exhibited favourable effects [50]. These compounds stimulated osteoblast proliferation, enhanced the OPG/RANKL ratio, and upregulated the expression of osteoblast differentiation markers. This heightened osteoblastic activity plays a crucial role in preventing alveolar bone loss and expediting mineralisation [51].

In all preclinical studies, a notable improvement in inflammation status was observed, characterised by significant reductions in ILs (IL-1β, IL-6 [33, 36], IL-8 [36], IL-10 [34]), TNF-α [34, 36], RANKL [31, 34], and nicotinamide adenine dinucleotide phosphate (NADPH) oxidase [33]. In addition, Gennaro et al. [34] noticed that green tea intake increased the expression of the osteogenesis-related factor RUNX-2 and the anti-osteoclastogenic factor OPG. Toker et al. [35] highlighted the significant decrease in matrix metalloproteinase-8 (MMP-8) and hypoxia-inducible factor-1 alpha (HIF-1α) levels with grape seed proanthocyanidin extract. Finally, Zhen et al. [36] reported that resveratrol administration significantly suppressed elevated toll-like receptor 4 (TLR4) levels in gingival tissue of mice. Targeting these inflammatory mediators and reducing their activity holds potential benefits for patients with chronic periodontitis. The decrease in the production of ILs and TNF-α contributes to the regression of bone resorption and soft tissue injury [52]. NADPH oxidase, responsible for generating ROS, which can cause oxidative stress in periodontal tissues, saw lowered activity. This reduction in NADPH oxidase activity could lead to decreased ROS levels, thereby mitigating oxidative stress and its detrimental effects on periodontal tissues [53].

The DM condition in animals also garnered attention. Zhen et al. [36] discovered that resveratrol significantly lowered blood glucose levels compared to untreated mice with experimental periodontitis. Similarly, insulin and resveratrol therapy [33], green tea intake [32, 34] reduced hyperglycaemia in diabetic mice. In contrast, Toker et al. [35] did not observe any significant difference in blood glucose levels among the diabetic groups during the study. Also, Alpan et al. [31] confirmed that taxifolin did not affect blood glucose levels in diabetic mice. Exploring diabetes monitoring through polyphenol supplementation should be expanded, particularly given the promising results observed in other preclinical studies regarding the insulin-sensitising properties of polyphenols [54, 55].

After carefully examining the included studies, it is apparent that the current evidence regarding the positive effects of polyphenols on periodontal and DM status, as well as their anti-inflammatory properties, remains insufficient. While some studies have shown promising results in improving periodontal health and DM parameters, it is essential to acknowledge the limitations of the current literature, such as the high risk of bias in some clinical trials and the limited evidence regarding diabetes condition after polyphenol supplementation in human studies. The experimental conditions and findings from animal studies cannot be directly applied to clinical practice, as the biological processes studied in animal models may not precisely replicate those occurring in the human body. For these reasons, experimental animal studies are typically situated at the base of the evidence hierarchy and are considered to have the lowest level of evidence when evaluating the applicability of findings to human health and clinical practice. Also, one of the drawbacks of this systematic review is the restricted quantity of clinical trials, compounded by the fact that all investigations were carried out by a single research group. The authors of the included clinical studies assessed periodontal status using parameters such as PD and CAL. The clear impact of polyphenol supplementation in humans cannot be fully evaluated due to the lack of comprehensive information regarding alveolar bone loss, bleeding on probing, the number of missing teeth, etc. To establish the true potential of polyphenols as an adjunctive treatment for periodontal diseases, it is crucial to conduct long-term, well-designed observational studies and clinical trials. These studies should employ rigorous methodologies and include larger sample sizes to ensure robust and reliable outcomes.

## Conclusion

The findings from this review preclude a definitive conclusion regarding the specific impact of polyphenols on periodontitis. Notably, the included preclinical studies provide substantial evidence to enhance our understanding of polyphenols’ effects, particularly in terms of reducing bone loss and improving glycaemic control. Further research and additional clinical studies may offer more comprehensive insights into the potential benefits of polyphenol supplementation in the context of periodontal health, especially in the presence of DM.

## Disclosure statement

The authors report there are no competing interests to declare.

## Data availability statement

Data related to the study are available upon request from via email to the corresponding author.

## Funding

No funding was obtained for this study.

## Geolocation information

Kaunas, Lithuania.
